# Bis[2-(1*H*-1,2,3-benzotriazol-1-yl)acetic acid-κ*N* ^3^]dichloridozinc(II)

**DOI:** 10.1107/S1600536808006399

**Published:** 2008-05-03

**Authors:** Tian Hang, Qiong Ye

**Affiliations:** a Ordered Matter Science Research Center, Southeast University, Nanjing 210096, People’s Republic of China

## Abstract

In the title complex, [ZnCl_2_(C_8_H_7_N_3_O_2_)_2_], the Zn^II^ atom is coordinated by two chloride ions and two N atoms in a distorted tetra­hedral coordination environment. In the crystal structure, mol­ecules are linked by inter­molecular C—H⋯O and O—H⋯O hydrogen bonds, forming a three-dimensional network.

## Related literature

For synthesis of the organic ligand, see: Danan *et al.* (1997 ▶); Xu & Ye (2007 ▶).
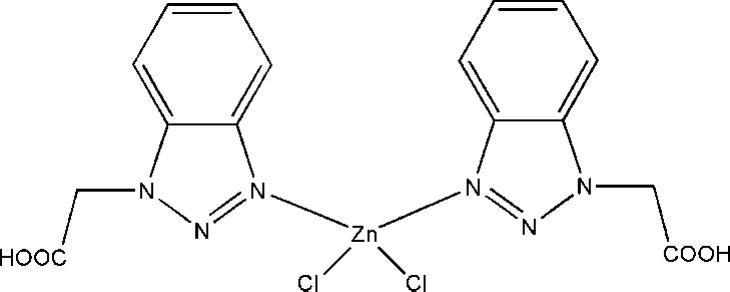


## Experimental

### 

#### Crystal data

[ZnCl_2_(C_8_H_7_N_3_O_2_)_2_]
*M* *_r_* = 490.60
Triclinic, 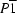

*a* = 8.0896 (16) Å
*b* = 9.6898 (19) Å
*c* = 12.703 (3) Å
α = 87.48 (3)°
β = 84.25 (3)°
γ = 83.95 (3)°
*V* = 984.7 (4) Å^3^
*Z* = 2
Mo *K*α radiation
μ = 1.55 mm^−1^
*T* = 293 (2) K
0.3 × 0.2 × 0.1 mm

#### Data collection

Rigaku Mercury2 CCD diffractometer
Absorption correction: multi-scan (*CrystalClear*; Rigaku, 2005 ▶) *T*_min_ = 0.720, *T*_max_ = 0.860
10222 measured reflections
4512 independent reflections
4022 reflections with *I* > 2σ(*I*)
*R*_int_ = 0.028

#### Refinement

*R*[*F* ^2^ > 2σ(*F* ^2^)] = 0.038
*wR*(*F* ^2^) = 0.099
*S* = 1.09
4512 reflections
275 parameters
2 restraints
H-atom parameters constrained
Δρ_max_ = 0.63 e Å^−3^
Δρ_min_ = −0.54 e Å^−3^

### 

Data collection: *CrystalClear* (Rigaku, 2005 ▶); cell refinement: *CrystalClear*; data reduction: *CrystalClear*; program(s) used to solve structure: *SHELXS97* (Sheldrick, 2008 ▶); program(s) used to refine structure: *SHELXL97* (Sheldrick, 2008 ▶); molecular graphics: *SHELXTL* (Sheldrick, 2008 ▶); software used to prepare material for publication: *SHELXTL*.

## Supplementary Material

Crystal structure: contains datablocks I, global. DOI: 10.1107/S1600536808006399/pk2083sup1.cif

Structure factors: contains datablocks I. DOI: 10.1107/S1600536808006399/pk2083Isup2.hkl

Additional supplementary materials:  crystallographic information; 3D view; checkCIF report

## Figures and Tables

**Table 1:** Hydrogen-bond geometry (Å, °)

*D*—H⋯*A*	*D*—H	H⋯*A*	*D*⋯*A*	*D*—H⋯*A*
O1—H1*C*⋯O4^i^	0.82	2.20	3.015 (4)	171
O2—H2*B*⋯Cl1^ii^	0.93	2.59	3.348 (3)	139
C6—H6*A*⋯O4^iii^	0.97	2.69	3.586 (4)	153

Symmetry codes: (i) 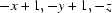; (ii) 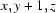; (iii) 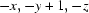.

**Fig. 1.**
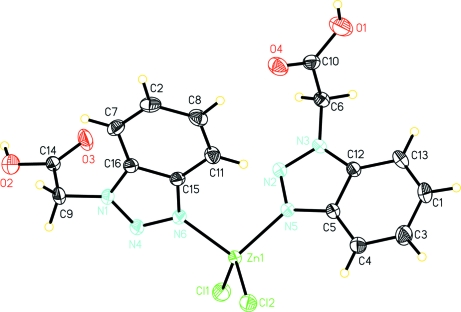
The molecular structure of the compound with the atomic numbering scheme. Displacement ellipsoids were drawn at the 30% probability level.

**Fig. 2.**
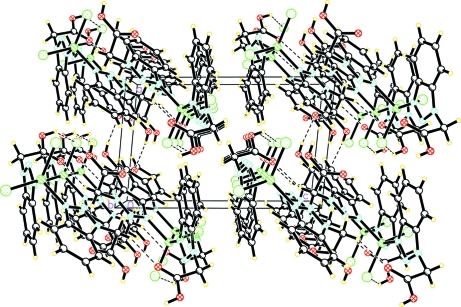
A packing diagram of the molecular packing of the title compound, viewed down the b axis.

## supplementary crystallographic information

### Comment

Recently, we reported the structure of 2-(1*H*-benzo[*d*][1,2,3] triazol-1-yl)acetonitrile (Xu *et al.* (2007)). The reaction of it with ZnCl_2_ in ethanol-water solution gives the title complex. The zinc(II) center is coordinated by two nitrogen atoms from the benzotriazole rings and two terminal chloride anions in a distorted tetrahedral arrangement as shown in Fig. 1. The angle between the two benzotriazole rings is 62.98 (7)°.

From Fig. 2, it is easy to see that the structure is consolidated by extensive C—H···O and O—H···O hydrogen bonds. This hydrogen bonding with the π-π stacking between neighboring results to the formation of three-dimensional structure.

### Experimental

The ligand, 2-(1*H*-benzo[*d*][1,2,3]triazol-1-yl)acetonitrile, was synthesized by the reaction of benzotriazole and bromoacetonitrile according to the procedure described in the literature (Danan *et al.* (1997)).

A mixture of 2-(1*H*-benzo[*d*][1,2,3]triazol-1-yl)acetonitrile (32 mg, 0.2 mmol), ZnCl_2_(40 mg, 0.3 mmol), ethanol(1 ml) and a few drops of water sealed in a glass tube maintained at 120 °C. Colorless crystals suitable for X-ray analysis were obtained after several days.

### Refinement

Positional parameters of all the H atoms were calculated geometrically and were allowed to ride on the C, O atoms to which they are bonded, with *U*_iso_(H) = 1.2*U*_eq_(C) or *U*_iso_(H) = 1.5*U*_eq_(O).

### Crystal data

**Table:**

[ZnCl_2_(C_8_H_7_N_3_O_2_)_2_]	*Z* = 2
*M**_r_* = 490.60	*F*_000_ = 496
Triclinic, *P*1	*D*_x_ = 1.655 Mg m^−^^3^
Hall symbol: -P 1	Mo *K*α radiation λ = 0.71073 Å
*a* = 8.0896 (16) Å	Cell parameters from 10272 reflections
*b* = 9.6898 (19) Å	θ = 3.1–28.7º
*c* = 12.703 (3) Å	µ = 1.56 mm^−^^1^
α = 87.48 (3)º	*T* = 293 (2) K
β = 84.25 (3)º	Block, colorless
γ = 83.95 (3)º	0.3 × 0.2 × 0.1 mm
*V* = 984.7 (4) Å^3^	

### Data collection

**Table:**

Mercury2 CCD (2x2 bin mode) diffractometer	4512 independent reflections
Radiation source: fine-focus sealed tube	4022 reflections with *I* > 2σ(*I*)
Monochromator: graphite	*R*_int_ = 0.028
Detector resolution: 13.6612 pixels mm^-1^	θ_max_ = 27.5º
*T* = 293(2) K	θ_min_ = 3.1º
CCD_Profile_fitting scans	*h* = −10→10
Absorption correction: multi-scan(CrystalClear; Rigaku, 2005)	*k* = −12→12
*T*_min_ = 0.720, *T*_max_ = 0.860	*l* = −16→16
10222 measured reflections	

### Refinement

**Table:**

Refinement on *F*^2^	Secondary atom site location: difference Fourier map
Least-squares matrix: full	Hydrogen site location: inferred from neighbouring sites
*R*[*F*^2^ > 2σ(*F*^2^)] = 0.038	H-atom parameters constrained
*wR*(*F*^2^) = 0.099	*w* = 1/[σ^2^(*F*_o_^2^) + (0.0423*P*)^2^ + 0.762*P*] where *P* = (*F*_o_^2^ + 2*F*_c_^2^)/3
*S* = 1.09	(Δ/σ)_max_ < 0.001
4512 reflections	Δρ_max_ = 0.63 e Å^−^^3^
275 parameters	Δρ_min_ = −0.54 e Å^−^^3^
2 restraints	Extinction correction: none
Primary atom site location: structure-invariant direct methods	

### Special details

**Table:**

Geometry. All e.s.d.'s (except the e.s.d. in the dihedral angle between two l.s. planes) are estimated using the full covariance matrix. The cell e.s.d.'s are taken into account individually in the estimation of e.s.d.'s in distances, angles and torsion angles; correlations between e.s.d.'s in cell parameters are only used when they are defined by crystal symmetry. An approximate (isotropic) treatment of cell e.s.d.'s is used for estimating e.s.d.'s involving l.s. planes.
Refinement. Refinement of *F*^2^ against ALL reflections. The weighted *R*-factor *wR* and goodness of fit *S* are based on *F*^2^, conventional *R*-factors *R* are based on *F*, with *F* set to zero for negative *F*^2^. The threshold expression of *F*^2^ > 2σ(*F*^2^) is used only for calculating *R*-factors(gt) *etc*. and is not relevant to the choice of reflections for refinement. *R*-factors based on *F*^2^ are statistically about twice as large as those based on *F*, and *R*- factors based on ALL data will be even larger.

### Fractional atomic coordinates and isotropic or equivalent isotropic displacement parameters (Å^2^)

**Table:**

	*x*	*y*	*z*	*U*_iso_*/*U*_eq_	
Zn1	−0.24666 (3)	0.21707 (3)	0.28538 (2)	0.03130 (10)	
Cl1	−0.49995 (9)	0.21702 (8)	0.23354 (6)	0.04842 (18)	
Cl2	−0.17619 (9)	0.06835 (7)	0.41738 (5)	0.04511 (17)	
O1	0.4086 (3)	0.4222 (3)	−0.1108 (2)	0.0764 (8)	
H1C	0.4884	0.4518	−0.0874	0.115*	
O2	−0.5545 (3)	0.9218 (2)	0.3737 (2)	0.0627 (6)	
H2B	−0.5859	0.9846	0.3201	0.061 (11)*	
O3	−0.3617 (3)	0.7988 (2)	0.26399 (16)	0.0555 (6)	
O4	0.2761 (3)	0.4739 (3)	0.05103 (16)	0.0546 (6)	
N1	−0.2805 (3)	0.6045 (2)	0.41091 (17)	0.0327 (4)	
N2	−0.0410 (3)	0.2952 (2)	0.09576 (17)	0.0350 (5)	
N3	0.0805 (3)	0.2522 (2)	0.02244 (16)	0.0338 (4)	
N4	−0.3401 (3)	0.4916 (2)	0.37794 (17)	0.0350 (5)	
N5	−0.0605 (3)	0.1913 (2)	0.16490 (16)	0.0334 (4)	
N6	−0.2121 (3)	0.4090 (2)	0.33810 (17)	0.0330 (4)	
C1	0.2918 (4)	−0.1020 (3)	0.0338 (2)	0.0470 (7)	
H1A	0.3714	−0.1660	0.0004	0.048 (9)*	
C2	0.1680 (4)	0.6422 (3)	0.3786 (2)	0.0465 (7)	
H2A	0.2506	0.6985	0.3893	0.061 (10)*	
C3	0.1993 (4)	−0.1432 (3)	0.1290 (2)	0.0504 (7)	
H3A	0.2219	−0.2330	0.1565	0.056 (10)*	
C4	0.0791 (4)	−0.0547 (3)	0.1816 (2)	0.0434 (6)	
H4A	0.0190	−0.0820	0.2439	0.048 (9)*	
C5	0.0517 (3)	0.0791 (3)	0.13594 (19)	0.0329 (5)	
C6	0.1357 (3)	0.3460 (3)	−0.0639 (2)	0.0380 (6)	
H6A	0.0439	0.4144	−0.0789	0.050 (9)*	
H6B	0.1698	0.2937	−0.1272	0.051 (9)*	
C7	0.0055 (4)	0.6881 (3)	0.4093 (2)	0.0404 (6)	
H7A	−0.0256	0.7737	0.4397	0.042 (8)*	
C8	0.2157 (3)	0.5130 (3)	0.3319 (2)	0.0456 (7)	
H8A	0.3282	0.4876	0.3121	0.054 (9)*	
C9	−0.3956 (3)	0.7224 (3)	0.4457 (2)	0.0375 (6)	
H9A	−0.3464	0.7727	0.4970	0.056 (10)*	
H9B	−0.4979	0.6900	0.4797	0.057 (10)*	
C10	0.2827 (4)	0.4195 (3)	−0.0345 (2)	0.0391 (6)	
C11	0.1012 (3)	0.4239 (3)	0.3143 (2)	0.0391 (6)	
H11A	0.1325	0.3381	0.2844	0.042 (8)*	
C12	0.1438 (3)	0.1188 (3)	0.0429 (2)	0.0323 (5)	
C13	0.2670 (3)	0.0281 (3)	−0.0107 (2)	0.0415 (6)	
H13A	0.3282	0.0552	−0.0727	0.044 (8)*	
C14	−0.4354 (3)	0.8186 (3)	0.3517 (2)	0.0360 (5)	
C15	−0.0664 (3)	0.4692 (2)	0.34439 (19)	0.0313 (5)	
C16	−0.1116 (3)	0.5972 (2)	0.39085 (19)	0.0311 (5)	

### Atomic displacement parameters (Å^2^)

**Table:**

	*U*^11^	*U*^22^	*U*^33^	*U*^12^	*U*^13^	*U*^23^
Zn1	0.03324 (16)	0.02833 (16)	0.03146 (16)	−0.00575 (11)	0.00609 (11)	−0.00602 (11)
Cl1	0.0373 (3)	0.0458 (4)	0.0624 (5)	−0.0034 (3)	−0.0060 (3)	−0.0035 (3)
Cl2	0.0551 (4)	0.0419 (4)	0.0363 (3)	−0.0022 (3)	0.0009 (3)	0.0019 (3)
O1	0.0671 (16)	0.100 (2)	0.0642 (16)	−0.0331 (15)	0.0161 (12)	−0.0134 (15)
O2	0.0577 (14)	0.0495 (13)	0.0762 (17)	0.0028 (10)	0.0081 (12)	−0.0038 (12)
O3	0.0670 (14)	0.0550 (13)	0.0375 (11)	0.0109 (11)	0.0131 (10)	−0.0053 (9)
O4	0.0568 (13)	0.0696 (15)	0.0398 (11)	−0.0209 (11)	0.0038 (10)	−0.0117 (10)
N1	0.0334 (10)	0.0284 (10)	0.0360 (11)	−0.0040 (8)	0.0012 (8)	−0.0081 (8)
N2	0.0393 (11)	0.0324 (11)	0.0317 (11)	−0.0044 (9)	0.0075 (9)	−0.0042 (8)
N3	0.0337 (11)	0.0345 (11)	0.0318 (11)	−0.0055 (8)	0.0071 (8)	−0.0037 (8)
N4	0.0323 (11)	0.0330 (11)	0.0400 (12)	−0.0074 (8)	0.0028 (9)	−0.0100 (9)
N5	0.0383 (11)	0.0279 (10)	0.0321 (11)	−0.0036 (8)	0.0067 (9)	−0.0025 (8)
N6	0.0320 (10)	0.0291 (10)	0.0384 (11)	−0.0068 (8)	0.0014 (9)	−0.0074 (8)
C1	0.0420 (15)	0.0468 (16)	0.0484 (16)	0.0110 (12)	0.0016 (12)	−0.0103 (13)
C2	0.0382 (14)	0.0512 (17)	0.0539 (17)	−0.0206 (12)	−0.0079 (13)	0.0027 (13)
C3	0.0575 (18)	0.0400 (16)	0.0492 (17)	0.0097 (13)	0.0000 (14)	0.0012 (13)
C4	0.0512 (16)	0.0376 (14)	0.0386 (14)	−0.0014 (12)	0.0051 (12)	0.0023 (11)
C5	0.0348 (12)	0.0324 (12)	0.0306 (12)	−0.0029 (10)	0.0031 (10)	−0.0053 (10)
C6	0.0429 (14)	0.0397 (14)	0.0303 (12)	−0.0063 (11)	0.0039 (10)	0.0002 (10)
C7	0.0463 (15)	0.0324 (13)	0.0450 (15)	−0.0125 (11)	−0.0068 (12)	−0.0032 (11)
C8	0.0297 (13)	0.0563 (18)	0.0498 (16)	−0.0046 (12)	0.0004 (12)	0.0011 (13)
C9	0.0386 (14)	0.0369 (13)	0.0352 (13)	0.0004 (11)	0.0051 (11)	−0.0115 (11)
C10	0.0445 (14)	0.0359 (13)	0.0364 (14)	−0.0084 (11)	0.0013 (10)	0.0022 (11)
C11	0.0335 (13)	0.0417 (14)	0.0411 (14)	−0.0010 (11)	−0.0007 (11)	−0.0055 (11)
C12	0.0304 (12)	0.0343 (13)	0.0319 (12)	−0.0045 (10)	0.0013 (9)	−0.0044 (10)
C13	0.0337 (13)	0.0514 (16)	0.0367 (14)	0.0003 (11)	0.0069 (11)	−0.0062 (12)
C14	0.0353 (13)	0.0332 (12)	0.0393 (14)	−0.0078 (9)	0.0051 (10)	−0.0086 (10)
C15	0.0325 (12)	0.0299 (12)	0.0318 (12)	−0.0064 (9)	−0.0015 (9)	−0.0019 (9)
C16	0.0326 (12)	0.0301 (12)	0.0310 (12)	−0.0061 (9)	−0.0014 (9)	−0.0015 (9)

### Geometric parameters (Å, °)

**Table:**

Zn1—N5	2.041 (2)	C2—C7	1.367 (4)
Zn1—N6	2.059 (2)	C2—C8	1.410 (4)
Zn1—Cl1	2.2142 (9)	C2—H2A	0.9295
Zn1—Cl2	2.2403 (10)	C3—C4	1.369 (4)
O1—C10	1.336 (4)	C3—H3A	0.9301
O1—H1C	0.8200	C4—C5	1.402 (4)
O2—C14	1.332 (3)	C4—H4A	0.9297
O2—H2B	0.9295	C5—C12	1.394 (3)
O3—C14	1.223 (3)	C6—C10	1.533 (4)
O4—C10	1.222 (3)	C6—H6A	0.9698
N1—N4	1.340 (3)	C6—H6B	0.9703
N1—C16	1.360 (3)	C7—C16	1.402 (3)
N1—C9	1.451 (3)	C7—H7A	0.9301
N2—N5	1.318 (3)	C8—C11	1.372 (4)
N2—N3	1.331 (3)	C8—H8A	0.9298
N3—C12	1.363 (3)	C9—C14	1.521 (4)
N3—C6	1.459 (3)	C9—H9A	0.9703
N4—N6	1.314 (3)	C9—H9B	0.9702
N5—C5	1.379 (3)	C11—C15	1.400 (4)
N6—C15	1.380 (3)	C11—H11A	0.9294
C1—C13	1.363 (4)	C12—C13	1.401 (4)
C1—C3	1.422 (4)	C13—H13A	0.9300
C1—H1A	0.9292	C15—C16	1.395 (3)
			
N5—Zn1—N6	101.73 (9)	N3—C6—H6A	109.6
N5—Zn1—Cl1	113.50 (7)	C10—C6—H6A	109.7
N6—Zn1—Cl1	110.90 (7)	N3—C6—H6B	109.5
N5—Zn1—Cl2	107.14 (7)	C10—C6—H6B	109.4
N6—Zn1—Cl2	104.41 (7)	H6A—C6—H6B	108.2
Cl1—Zn1—Cl2	117.63 (4)	C2—C7—C16	115.2 (3)
C10—O1—H1C	109.5	C2—C7—H7A	122.6
C14—O2—H2B	119.5	C16—C7—H7A	122.3
N4—N1—C16	111.2 (2)	C11—C8—C2	122.0 (3)
N4—N1—C9	119.7 (2)	C11—C8—H8A	119.0
C16—N1—C9	128.3 (2)	C2—C8—H8A	119.0
N5—N2—N3	107.4 (2)	N1—C9—C14	110.2 (2)
N2—N3—C12	111.6 (2)	N1—C9—H9A	109.7
N2—N3—C6	120.6 (2)	C14—C9—H9A	109.7
C12—N3—C6	127.7 (2)	N1—C9—H9B	109.6
N6—N4—N1	107.55 (19)	C14—C9—H9B	109.5
N2—N5—C5	109.6 (2)	H9A—C9—H9B	108.2
N2—N5—Zn1	118.20 (16)	O4—C10—O1	125.3 (3)
C5—N5—Zn1	132.12 (17)	O4—C10—C6	120.4 (2)
N4—N6—C15	109.65 (19)	O1—C10—C6	114.2 (2)
N4—N6—Zn1	120.39 (16)	C8—C11—C15	116.2 (3)
C15—N6—Zn1	129.87 (17)	C8—C11—H11A	122.1
C13—C1—C3	122.2 (3)	C15—C11—H11A	121.6
C13—C1—H1A	118.7	N3—C12—C5	104.5 (2)
C3—C1—H1A	119.1	N3—C12—C13	133.1 (2)
C7—C2—C8	122.8 (3)	C5—C12—C13	122.4 (2)
C7—C2—H2A	118.6	C1—C13—C12	115.8 (3)
C8—C2—H2A	118.6	C1—C13—H13A	122.1
C4—C3—C1	122.1 (3)	C12—C13—H13A	122.1
C4—C3—H3A	119.1	O3—C14—O2	124.5 (3)
C1—C3—H3A	118.8	O3—C14—C9	120.8 (2)
C3—C4—C5	116.1 (3)	O2—C14—C9	114.6 (2)
C3—C4—H4A	121.8	N6—C15—C16	106.8 (2)
C5—C4—H4A	122.1	N6—C15—C11	132.0 (2)
N5—C5—C12	106.9 (2)	C16—C15—C11	121.1 (2)
N5—C5—C4	131.7 (2)	N1—C16—C15	104.7 (2)
C12—C5—C4	121.4 (2)	N1—C16—C7	132.6 (2)
N3—C6—C10	110.5 (2)	C15—C16—C7	122.7 (2)
			
N5—N2—N3—C12	−0.4 (3)	C16—N1—C9—C14	81.9 (3)
N5—N2—N3—C6	−177.9 (2)	N3—C6—C10—O4	−48.2 (4)
C16—N1—N4—N6	1.1 (3)	N3—C6—C10—O1	134.4 (3)
C9—N1—N4—N6	171.8 (2)	C2—C8—C11—C15	0.6 (4)
N3—N2—N5—C5	0.4 (3)	N2—N3—C12—C5	0.3 (3)
N3—N2—N5—Zn1	−176.82 (16)	C6—N3—C12—C5	177.6 (2)
N6—Zn1—N5—N2	−47.2 (2)	N2—N3—C12—C13	178.1 (3)
Cl1—Zn1—N5—N2	72.01 (19)	C6—N3—C12—C13	−4.6 (5)
Cl2—Zn1—N5—N2	−156.44 (17)	N5—C5—C12—N3	0.0 (3)
N6—Zn1—N5—C5	136.3 (2)	C4—C5—C12—N3	178.8 (2)
Cl1—Zn1—N5—C5	−104.5 (2)	N5—C5—C12—C13	−178.2 (2)
Cl2—Zn1—N5—C5	27.1 (2)	C4—C5—C12—C13	0.7 (4)
N1—N4—N6—C15	−0.4 (3)	C3—C1—C13—C12	−0.4 (4)
N1—N4—N6—Zn1	176.54 (15)	N3—C12—C13—C1	−177.6 (3)
N5—Zn1—N6—N4	148.39 (19)	C5—C12—C13—C1	−0.1 (4)
Cl1—Zn1—N6—N4	27.4 (2)	N1—C9—C14—O3	−6.9 (4)
Cl2—Zn1—N6—N4	−100.26 (19)	N1—C9—C14—O2	172.8 (2)
N5—Zn1—N6—C15	−35.3 (2)	N4—N6—C15—C16	−0.4 (3)
Cl1—Zn1—N6—C15	−156.3 (2)	Zn1—N6—C15—C16	−176.95 (17)
Cl2—Zn1—N6—C15	76.0 (2)	N4—N6—C15—C11	179.4 (3)
C13—C1—C3—C4	0.3 (5)	Zn1—N6—C15—C11	2.8 (4)
C1—C3—C4—C5	0.3 (5)	C8—C11—C15—N6	179.5 (3)
N2—N5—C5—C12	−0.2 (3)	C8—C11—C15—C16	−0.8 (4)
Zn1—N5—C5—C12	176.47 (18)	N4—N1—C16—C15	−1.3 (3)
N2—N5—C5—C4	−178.9 (3)	C9—N1—C16—C15	−170.9 (2)
Zn1—N5—C5—C4	−2.2 (4)	N4—N1—C16—C7	179.4 (3)
C3—C4—C5—N5	177.8 (3)	C9—N1—C16—C7	9.7 (5)
C3—C4—C5—C12	−0.7 (4)	N6—C15—C16—N1	1.0 (3)
N2—N3—C6—C10	92.5 (3)	C11—C15—C16—N1	−178.8 (2)
C12—N3—C6—C10	−84.5 (3)	N6—C15—C16—C7	−179.6 (2)
C8—C2—C7—C16	−0.1 (4)	C11—C15—C16—C7	0.6 (4)
C7—C2—C8—C11	−0.1 (5)	C2—C7—C16—N1	179.1 (3)
N4—N1—C9—C14	−87.0 (3)	C2—C7—C16—C15	−0.1 (4)

### Hydrogen-bond geometry (Å, °)

**Table:**

*D*—H···*A*	*D*—H	H···*A*	*D*···*A*	*D*—H···*A*
O1—H1C···O4^i^	0.82	2.20	3.015 (4)	171
O2—H2B···Cl1^ii^	0.93	2.59	3.348 (3)	139
C6—H6A···O4^iii^	0.97	2.69	3.586 (4)	153

Symmetry codes: (i) −*x*+1, −*y*+1, −*z*; (ii) *x*, *y*+1, *z*; (iii) −*x*, −*y*+1, −*z*.
